# Vitamin D and the Athlete: Current Perspectives and New Challenges

**DOI:** 10.1007/s40279-017-0841-9

**Published:** 2018-01-24

**Authors:** Daniel J. Owens, Richard Allison, Graeme L. Close

**Affiliations:** 1 Research Institute for Sport and Exercise Science, Liverpool John Moores University, Tom Reilly Building, Byrom Street, Liverpool, L3 3AF UK; 2 Exercise and Sport Science Department, ASPETAR, Orthopaedic and Sports Medicine Hospital, Doha, Qatar; 3 Arsenal Football Club, Bell Lane, London Colney, St Albans, Shenley AL2 1DR UK

## Abstract

The last decade has seen a dramatic increase in general interest in and research into vitamin D, with many athletes now taking vitamin D supplements as part of their everyday dietary regimen. The most recognized role of vitamin D is its regulation of calcium homeostasis; there is a strong relationship between vitamin D and bone health in non-athletic individuals. In contrast, data have consistently failed to demonstrate any relationship between serum 25[OH]D and bone health, which may in part be due to the osteogenic stimulus of exercise. Vitamin D may interact with extra-skeletal tissues such as muscle and the immune system to modulate recovery from damaging exercise and infection risk. Given that many athletes now engage in supplementation, often consuming extreme doses of vitamin D, it is important to assess whether excessive vitamin D can be detrimental to health. It has been argued that toxic effects only occur when serum 25[OH]D concentrations are greater than 180 nmol·l^−1^, but data from our laboratory have suggested high-dose supplementation could be problematic. Finally, there is a paradoxical relationship between serum 25[OH]D concentration, ethnicity, and markers of bone health: Black athletes often present with low serum 25[OH]D without physiological consequences. One explanation for this could be genetic differences in vitamin D binding protein due to ethnicity, resulting in greater concentrations of bioavailable (or free) vitamin D in some ethnic groups. In the absence of any pathology, screening may be unnecessary and could result in incorrect supplementation. Data must now be re-examined, taking into consideration bioavailable or “free” vitamin D in ethnically diverse groups to enable new thresholds and target concentrations to be established; perhaps, for now, it is time to “set vitamin D free”.

## A Brief Historical Perspective

Vitamin D was first identified in the early twentieth century by a forerunner of nutritional biochemistry, McCollum [1]. His pioneering work on experimental rickets was the first to identify the existence of a vitamin that was responsible for calcium deposition [1], later called vitamin D. Primarily because of McCollum’s work and the rickets epidemic of the same era, vitamin D was long recognized only for its role in bone health. Studies that followed determined the main source of vitamin D as ultraviolet B (UVB) radiation exposure and showed that limited quantities could be obtained from the diet alone. This highlighted that the vitamin D endocrine system likely developed as an evolutionary adaptation to the sun-rich environments in which humans evolved.

With advancement of new technologies, our understanding of the vitamin D endocrine system and its biological significance has grown exponentially. Generation of a vitamin D receptor knockout mouse [2] and high-throughput gene microarrays have provided a bounty of newly identified vitamin D targets in numerous tissues such as bone [3, 4], immune system cells [5], the cardiovascular system [6], and skeletal muscle [7, 8]. Historically known for its canonical role in mediating bone turnover, the validated functions of vitamin D are now understood to be much further reaching. Many of the newly identified functions of vitamin D have relevance for athletic performance and, as such, vitamin D has found the spotlight in the world of sports nutrition. Optimizing muscle function and remodeling, maintaining bone health, and minimizing infection risk are key examples of how vitamin D may benefit the athlete. However, vitamin D is clearly not an ergogenic aid but a biological requirement, and therefore the use of exogenous vitamin D in any population is a function of vitamin D status. This message has unfortunately been lost in the search for marginal gains. As we will uncover, the process of categorizing vitamin D concentrations has led to confusion, and new evidence suggests that such guidelines may indeed be based upon the measurement of the wrong form of vitamin D.

The aim of this review is to provide a current perspective on the functions of vitamin D that may influence athletic performance, to clarify current guidelines for vitamin D intake, and to highlight crucial aspects of future work that must be tackled. We will address key areas of common confusion that surround vitamin D in the sports world, such as defining and measuring vitamin D and what physiological functions relevant to athletic performers can be optimized by maintenance of adequate vitamin D status.

## What is Vitamin D Deficiency?

**Fig. 1:**
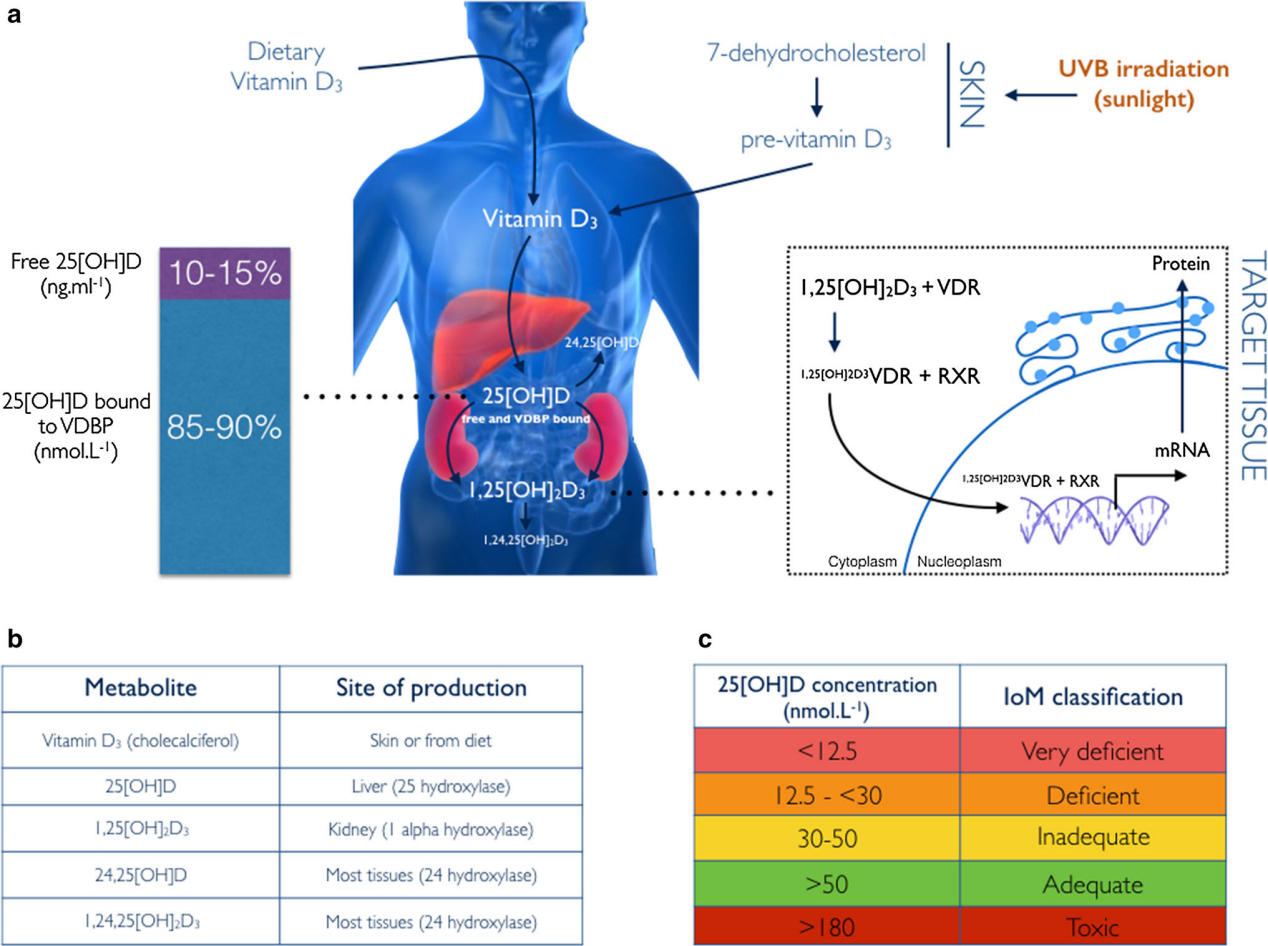
**a** Dietary vitamin D_3_ or exposure of skin to ultraviolet B (UVB) radiation results in circulating vitamin D_3_ (cholecalciferol). This metabolite is hydroxylated in the liver at carbon 25 to form the metabolite 25[OH]D, a biologically inactive compound with the longest half-life of the vitamin D metabolites. 25[OH]D circulates bound to vitamin D-binding protein (VDBP; 85–90%), whereas a smaller fraction circulates freely in serum (10–15%). 25[OH]D is transported to the kidney or target tissues expressing 1α-hydroxylase, where it is hydroxylated further at carbon 1 to form 1α,25[OH]D_2_D_3_, the biologically active vitamin D metabolite. At the target tissue, 1α,25[OH]D_2_D_3_ binds to the vitamin D receptor (VDR) and subsequently forms a heterodimer with retinoid X receptor (RXR), forming a transcriptional complex that recruits co-activators and repressors to vitamin D response elements to activate and repress the gene. **b** The most common vitamin D metabolites and their sites of production. **c** The US Institute of Medicine (IoM) guidelines for the classification of vitamin D status. *mRNA* messenger RNA

Exactly what constitutes vitamin D deficiency is subject to intense debate. Moreover, a lack of understanding of the key metabolites in the vitamin D pathway can lead to erroneous recommendations [9]. For this reason, we first present a collated overview of the US Institute of Medicine (IoM) guidelines for vitamin D classification [9] and a simplified schematic of the key vitamin D metabolites and sites of their production (Fig. 1).

The IoM guidelines refer to concentrations of total serum 25[OH]D, the sum of 25[OH]D_2_ and 25[OH]D_3_, which are biologically inactive metabolites produced by hydroxylation of the vitamin D_2_ and D_3_ precursors (Fig. 1). Although the parent sterol vitamin D has a half-life close to 24 h [10], this is relatively short compared with 25[OH]D with a half-life of 21–30 days [11]. The measurement of circulating 25[OH]D is a better indicator of vitamin D exposure, whether obtained from UVB exposure (contributing 80–90% of 25[OH]D) or dietary sources (contributing 10–20%). Measurements of these metabolites are typically made by extracting serum from a venous blood sample and eluting the vitamins before analysis via one of several methods. It is beyond the scope of this review to critique the analytical techniques for vitamin D metabolite measurement, but the current gold standard measurement is liquid chromatography tandem mass spectrometry (LC–MS/MS) [12].

It should also be considered that the US IoM reference intakes might be outdated. Recent commentaries clearly state that the IoM guidelines were developed based on the estimated average requirement (EAR) for vitamin D centered on its role in bone health [13]. Although the same authors state that extra-skeletal roles of vitamin D remain under study, a large evidence base of well-controlled trials suggests the regulation of bone turnover is but one functional role for vitamin D. At present, the most sensible approach is to avoid severe deficiency and concentrations considered toxic by the US IoM because the serum concentrations necessary to satisfy all biological needs with a vitamin D requirement is not yet fully understood.

More importantly, as we explore in more detail, the correlation between 25[OH]D and bone health is grossly misleading. Although it is unequivocal that vitamin D is required for calcium deposition and that 25[OH]D has been the best marker of identifying vitamin D exposure, consideration for what fraction of total 25[OH]D is measured has been largely overlooked for many years [14]. Moreover, some parameters of health correlate better to other metabolites of vitamin D [15]. This raises new questions as to whether measuring 25[OH]D to characterize vitamin D status is indeed the best practice.

## Supplemental Vitamin D: The “On Trend” in Sports Nutrition

The misinterpretation of medical guidelines and suggestions of new, non-validated sets of guidelines (such as those suggested by Heaney and Holick [16] and Zittermann [17]) have led to blanket supplemental vitamin D plans in elite sport becoming commonplace. It is reasonable to ask whether supplementation is necessary for all athletes.

Many studies have assessed 25[OH]D concentrations across the world in elite and sub-elite athletes throughout different months of the year [18–28]. Large variations exist between cohorts of non-supplemented athletes. For example, our laboratory has shown large variations between cohorts of elite rugby players, footballers, and jockeys [29]. Numerous factors, such as dietary differences, sunlight exposure, clothing, and lifestyle, may all contribute to the disparities [30]. It is important that athletes at risk of being deficient are tested before proceeding to correct the vitamin D inadequacy. It is crucial that applied practitioners and scientists are aware that whether athletes should be supplemented is purely based on whether they have sufficient or insufficient/deficient vitamin D concentrations. There is no ergogenic effect of providing doses of supplemental vitamin D that would elevate 25[OH]D concentrations far above the cut-off for sufficiency (> 75 nmol·l^−1^).

When a need to supplement has been identified, an appropriately screened supplemental form of vitamin D_3_ should be sourced that can deliver the correct dose. Recommendations regarding supplementation dose vary widely and can often be confusing. From the authors’ combined applied experience, dosing strategies for vitamin D in elite sport range from 1000 IU/day to blanket supplementation of up to a 100,000 IU bolus per week. This review should serve to direct practitioners towards a need for a supplementation decision system that should be implemented on an individual basis and provide the most current advice for safe and effective supplementation protocols.

## Functional Roles of Vitamin D Relevant to the Athlete

### Muscle Repair and Remodeling

The purpose of athletic training is to provide a stimulus that disrupts homeostasis to bring about an adaptive response that improves competition performance. For athletes, maximizing the training stimulus is therefore a core principle of the training program. Nutrition strategies to complement the adaptive response to a physical/metabolic challenge are intensely researched. Recently, on the basis of animal trials and in vitro basic biology studies, data have emerged suggestive of a beneficial role for vitamin D in skeletal muscle repair and remodeling.

In a randomized controlled trial (RCT), our laboratory showed that elevating serum 25[OH]D concentrations to > 75 nmol·l^−1^ with supplemental vitamin D_3_ at 4000 IU/day has a positive effect on the recovery of force following a bout of damaging eccentric exercise [31]. Similar results were observed in correlative studies between serum 25[OH]D and force recovery following intense exercise [32, 33]. These results imply that adequate vitamin D exposure can optimize the acute adaptive response to damaging physical work but do not lend any support to the idea that vitamin D may be important over an extended period of training. However, a recent training study provided evidence to support this idea [34]. The authors supplemented 40 untrained young and elderly men with vitamin D_3_ 1920 IU (48 μg) + 800 mg calcium per day during December–April (*n* = 20 per group), or calcium alone (placebo group) at a latitude of 56°N (very little sunlight exposure). During the final 12 weeks of the supplementation period, participants underwent a resistance training program for the quadriceps muscles. There were no observable differences between groups in strength gains or hypertrophy, but a great fiber type switch (more type IIA fibers) and a reduction in myostatin messenger RNA (mRNA) expression were observed in the young men receiving vitamin D. Interestingly, the elderly men receiving vitamin D showed an improvement in muscle quality above that of the placebo group. Taking in vivo data together, it appears that, where more drastic remodeling is required, perhaps with a requirement for satellite cell recruitment, vitamin D may exert more pronounced benefits in muscle.

**Fig. 2:**
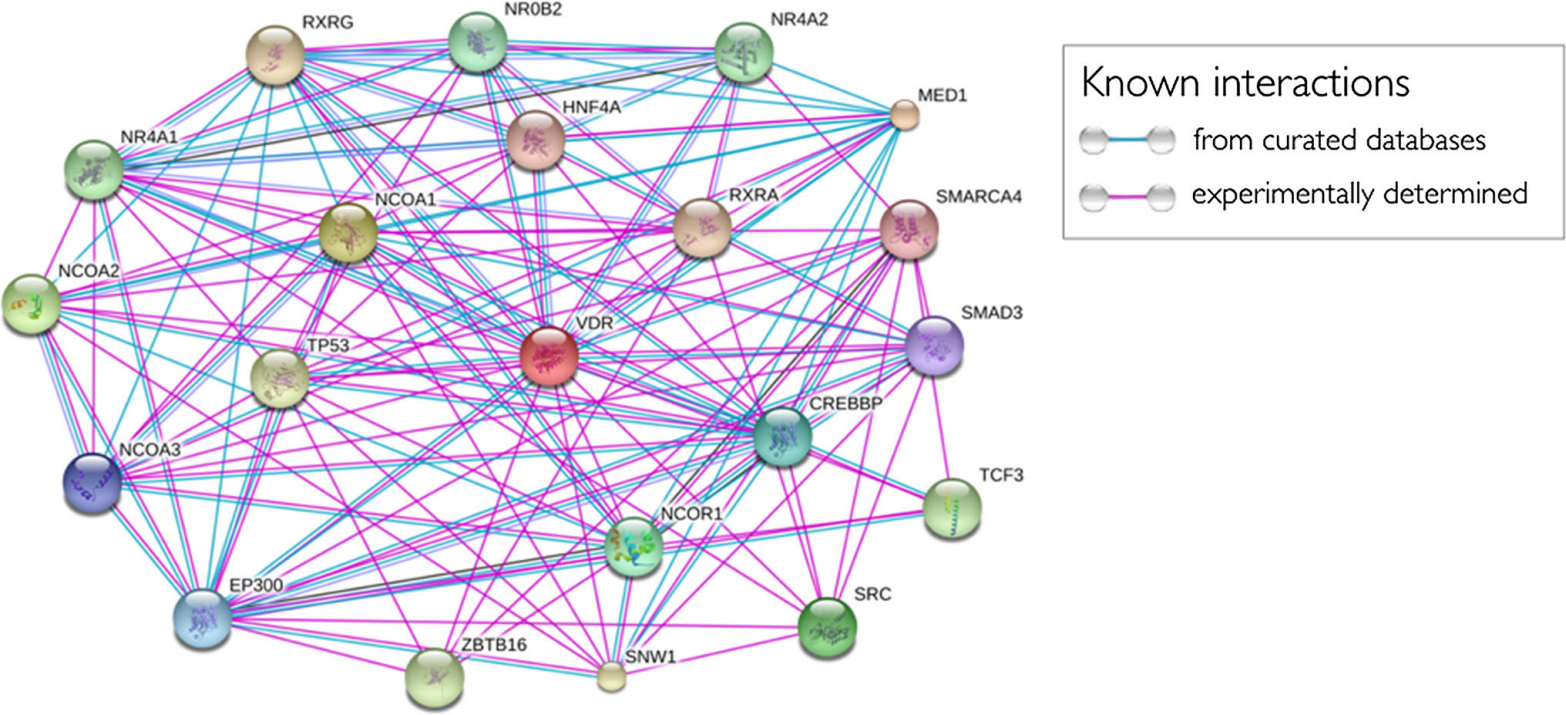
Known and predicted vitamin D receptor (VDR) protein interactions in *Homo sapiens*. The figure demonstrates numerous signaling pathways in which the VDR is involved. Each node (sphere) represents all the proteins produced by a single protein-coding gene locus. Lines connecting nodes represent the type of interaction, defined in the key. Note that interactions do not necessarily mean a physical interaction between proteins. Interactions were limited to no more than 20 interactions. The minimum known interaction score was set at 0.150 (http://string-db.org)

To definitively infer that vitamin D interacts with muscle to modulate some aspects of muscle remodeling, molecular mechanisms are essential. More studies have focused on the molecular actions of vitamin D in muscle than have focused on translational study designs, so the challenge to the field remains to decipher the key vitamin D targets in play during the remodeling process. One study analyzed global gene expression profiles during mechanical overload-induced skeletal muscle hypertrophy in the adult mouse [35]. Interestingly, the vitamin D receptor (VDR)/retinoid X receptor (RXR) nuclear receptor-signaling pathway showed significant upregulation during the early stages of hypertrophy. Given the known protein interactions of the VDR (presented graphically in Fig. 2), it is clear that VDR signaling interacts with pathways associated with the maintenance of skeletal muscle mass. In particular, it may be postulated that VDR signaling is important for satellite cell activity, consistent with in vivo observations discussed earlier. Emerging experimental evidence is in support of this notion. First, the VDR is expressed in satellite cells and can regulate cell fate decisions (i.e., to differentiate or divide and maintain the stem cell pool) in satellite cell cultures [36]. Moreover, downregulation of the notch pathway, a key regulator of satellite cell activation [37], has been reported in vitamin D-deficient myogenic cell cultures [38]. We have shown that the migration and fusion of human-derived skeletal muscle precursor cells is improved in the presence of 1,25[OH]_2_D_3_ [31]. mRNA and protein expression of the VDR appears higher in satellite cells than in mature muscle fibers, suggesting a more prominent role in muscle progenitors [36].

The signaling axes through which the VDR might mediate these effects are not well defined. However, the known and predicted VDR-interacting partners (presented in Fig. 2), such as mothers against decapentaplegic homolog 3 (smad3) (implicating the bone morphogenetic protein (BMP)/transforming growth factor (TGF)-β axis), Src (Proto-oncogene tyrosine-protein kinase Src)/phosphoinositide 3 kinase (PI3K), and cAMP responsible element-binding protein (CREB) cascades, are attractive candidates given their identified roles in muscle progenitor differentiation and regeneration of muscle following damage [39, 40].

In summary, both large-scale gene expression trials and focused experimental studies performed in vitro and in vivo suggest that vitamin D has the capacity to influence skeletal muscle remodeling, which is of importance to the athletic performer. Future studies could use publicly available gene and protein array data to identify candidate pathways for validation, through which vitamin D may exert its effects in satellite cells and potentially mature skeletal muscle fibers.

### Muscle Function

Whether vitamin D has the capacity to have any measurable effect on skeletal muscle function in young, trained athletes is debatable. Available data on this topic are limited and highly underpowered in young athletic populations. Moreover, the data that exist are mixed, with some reporting a positive effect of vitamin D [29, 41] and others reporting no effect [42, 43]. Many previous reviews discuss the role of vitamin D in muscle function of the athlete but discuss data from non-athletic populations. Athletes typically have minimal margins for improvement because they are highly trained. Thus, only directly observing effects in highly trained, athletic populations can give meaningful results that relate to high performance.

Assertions that vitamin D is important for muscle function may be due to consistently positive findings in elderly populations. By meta-analysis, it has been reported that a small number of studies demonstrate an increase in proximal muscle strength in adults with 25[OH]D concentrations < 25 nmol·l^−1^ [44]. It may be that the sarcopenic status of elderly muscle permits more measurable benefits to be gleaned from the maintenance of adequate vitamin D concentration. Another theory is that muscle function may only be perturbed with severe vitamin D deficiency, which is more prevalent in the ageing population [45].

It may be that vitamin D deficiency negatively impacts muscle function; however, data coupling cases of severe deficiency with muscle function in elite athletes do not exist. Therefore, at present it is not possible to suggest that vitamin D does play a role in the contractile properties and force-producing capacity of muscle in athletes. Large-scale RCTs are needed to address this question, and examination of athletes at the lowest end of vitamin D deficiency (< 25 nmol·l^−1^) is required.

### Innate and Acquired Immunity

Vitamin D has been reported to play important roles in aspects of both innate and acquired immune function [46, 47]. As stated previously (Fig. 1), the enzyme 1-alpha hydroxylase is responsible for the hydroxylation of the inactive 25[OH]D to its biologically active form, 1,25[OH]D. Also, the fact that monocytes, macrophages, neutrophils, and T and B lymphocytes contain not only the VDR but also 1-alpha hydroxylase suggests that vitamin D is functionally important to the immune system.

Activation in immune cells appears to be regulated by circulating concentrations of 25[OH]D and induced by activation of the toll-like receptor cascade in the presence of pathogenic microbiota [48]. In the immune system specifically, vitamin D upregulates gene expression of broad-spectrum anti-microbial peptides (AMPs), which are important regulators in innate immunity [49, 50]. Vitamin D also exerts an immunomodulatory effect on T and B lymphocytes in acquired immunity [17, 46]. AMPs, including cathelicidin, are important proteins in the innate immune system [51] and help defend against acute illness, including tuberculosis, influenza, and the common cold [52–54]. Vitamin D is further suggested to maintain a balance between the inflammatory type 1 and type 17 T-helper (TH1/TH17) cells and the immunosuppressive Th2/regulatory T cells (Tregs) to dampen excessive inflammation and tissue damage [55] and modulate the acquired immune response. Additional studies suggest that vitamin D enhances natural killer cell cytolytic activity [56] and acts to trigger the oxidative burst in activated macrophages [57]. A single dose of vitamin D_3_ (100,000 IU) has been shown to enhance the innate immune response and restrict growth of mycobacteria in vitro [57].

Variations in vitamin D concentrations have the potential to measurably influence the immune response. A handful of studies in athletes [46, 58], military personnel [59], and the general population [60–62] have reported negative associations between vitamin D concentration and incidences of upper respiratory tract infections (URTIs). In one study in college athletes, vitamin D concentrations over the winter and spring were negatively associated with documented frequency of acute URTI [63]. The breakpoint for contracting a single illness appeared to occur at ~ 95 nmol·l^−1^, such that all athletes with circulating concentrations lower than this breakpoint had one or more episodes of illness. Those with higher concentrations had one or fewer episodes. A similar study in endurance athletes reported that a greater proportion of athletes maintaining circulating 25[OH]D concentration < 30 nmol·l^−1^ presented with URTI symptoms, with the fewest symptoms reported in those with 25(OH)D concentrations > 120 nmol·l^−1^ [64]. Athletes with low vitamin D concentrations also had higher URTI symptom days and higher symptom-severity scores. However, randomly assigned placebo-controlled studies are needed in athletic populations to confirm the effectiveness of correcting low vitamin D concentrations on aspects of immune health and the prevention of URTIs. One recent RCT in university athletes found evidence that 14-week supplementation with vitamin D_3_ 5000 IU per day during winter training significantly increased salivary secretion rates of cathelicidin and secretory immunoglobulin A compared with a placebo control, which could improve resistance to respiratory infections [65].

### Cardiac Structure and Function

The heart and vascular system, like skeletal muscle, contain the VDR and the apparatus for 1,25[OH]_2_D_3_ production [66, 67]. An association between vitamin D concentration and cardiovascular function was first observed 30 years ago in Sprague–Dawley rats; histological analysis of vitamin D-deficient rats showed significantly smaller ventricular myofibrils and increases in extracellular matrix proteins compared with vitamin D-sufficient rats [67]. Subsequent research has established that vitamin D deficiency may adversely affect cardiac contractility, vascular tone, cardiac collagen content, and cardiac tissue maturation [68].

Human trials have produced some evidence that vitamin D deficiency may be related to an increased risk of cardiometabolic outcomes. The Framingham Offspring Study found an association between lower serum 25[OH]D concentrations and increased risk of cardiovascular events [50]. There was a graded increase in cardiovascular risk across pre-specified thresholds of 25[OH]D deficiency, with multivariable-adjusted hazard ratios of 1.53 (95% confidence interval (CI) 1.00–2.36) for levels 25 to < 37 nmol·l^−1^ and 1.80 (95% CI 1.05–3.08) for levels < 25 nmol·l^−1^. However, this relationship was found only among participants who were hypertensive at baseline [50]. Data from the HPFS (Health Professionals Follow-up Study) found a significant correlation between lower serum 25[OH]D concentration (defined as < 37 nmol·l^−1^) and elevated risk of myocardial infarction [69]. Scragg et al. [70] also showed an inverse relationship between 25[OH]D and incidence of myocardial infarction in the general population, although this association may have been intermediated by physical activity. In both the Tromso and Hoorn studies [71, 72], serum 25[OH]D was not associated with left ventricular (LV) structure and function, whereas later research reported that serum concentrations of 25[OH]D are significantly associated with LV diastolic dysfunction [73]. The relationship between vitamin D and cardiac function in the general population therefore remains controversial, with research generating equivocal evidence. The heterogeneity of research findings may be a consequence of differing definitions of vitamin D status, age of sample population, definition and determination of cardiovascular endpoints, and other confounding factors.

**Fig. 3:**
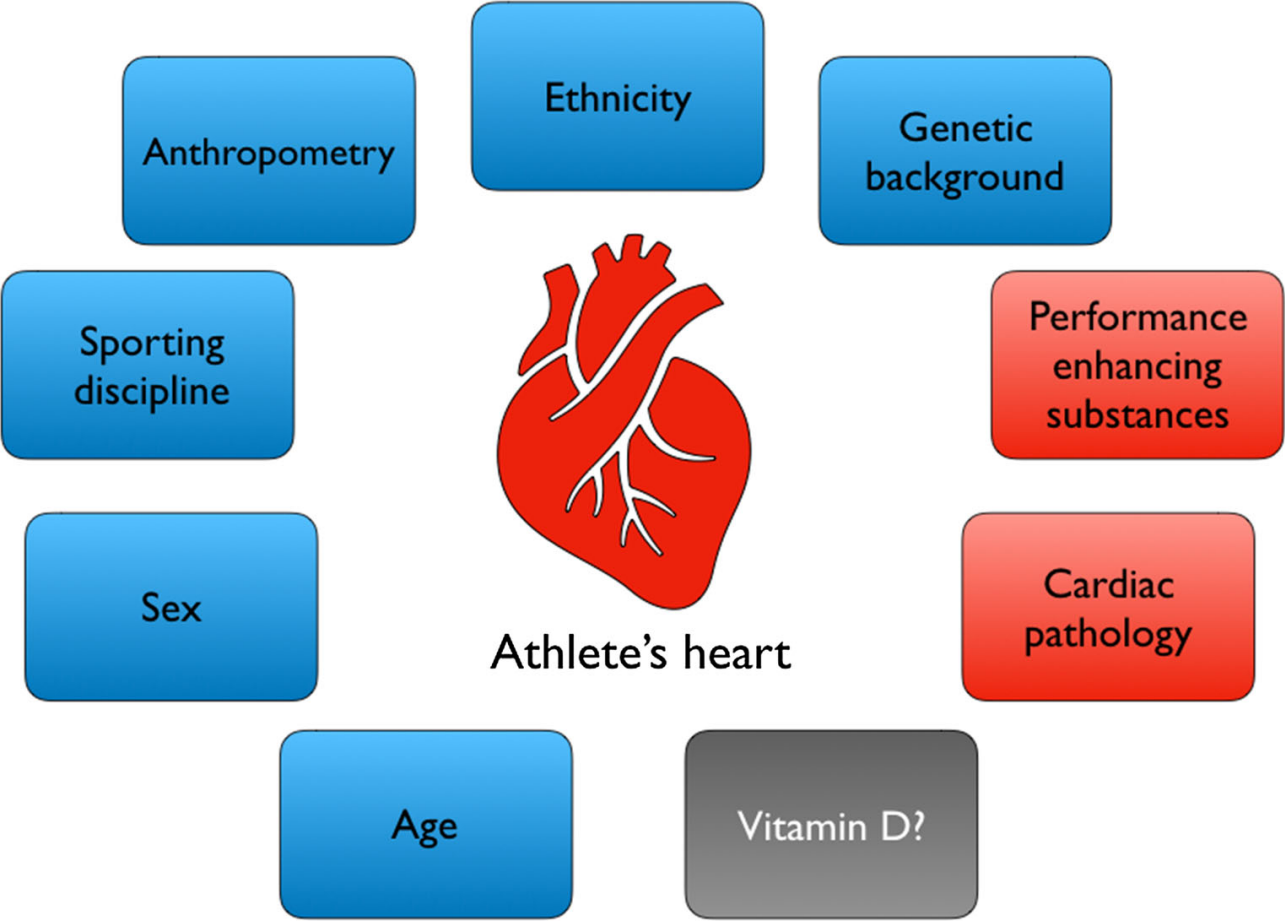
Schematic representation of demographic (*blue*) and pathological (*red*) factors that may influence the cardiovascular adaptation to exercise [133]

Professional athletes are unique amongst the general population, as they regularly participate in prolonged and intensive physical exercise that is associated with several structural and electrophysiological cardiac adaptations [74]. These adaptations enhance diastolic filling and facilitate a sustained increase in cardiac output, which is fundamental to athletic performance. Such cardiac adaptations are collectively referred to as the “athlete’s heart”. Numerous factors affect the adaptations of the athlete’s heart, including sporting modality, duration and intensity, age, ethnicity, sex, anthropometry, and performance-enhancing substance abuse (Fig. 3). Despite some evidence of a relationship between vitamin D and cardiac function, few studies have examined the association between 25[OH]D concentration and cardiac structure and function in healthy athletes, a population repeatedly reported to be vitamin D deficient [22, 29, 75]. Our group recently observed that the aortic root and left atria diameters, intraventricular septum diameter, LV diameter during diastole, LV mass, LV volume during diastole, and right atrial area of severely 25[OH]D-deficient (defined as < 25 nmol·l^−1^) athletes were significantly smaller than those of insufficient and sufficient athletes [76].

The precise mechanisms causing this lack of cardiac hypertrophy in the 25[OH]D-deficient state remain unclear. However, what is understood, is that the remodeling mechanisms associated with cardiac disease and chronic overloading (such as long-standing mitral insufficiency, essential hypertension, chronic heart failure, kidney disease, and dilated cardiomyopathy) differ considerably from the physiological adaptations seen in athletes induced through prolonged and intensive exercise.

Studies in rodent models show that the VDR and 1α-hydroxylase mediate arterial hardening and endothelial function. Also, the endothelial dysfunction observed in VDR-knockout mice is caused by a reduction in nitric oxide bioavailability [77]. Furthermore, vitamin D induces a counter-regulatory process in the renin-angiotensin-aldosterone system by diminishing its proliferating effects on the vascular smooth muscle cells [78]. There may also be vitamin D-dependent cardio-protective mechanisms, including reducing vessel wall damage caused by inflammation through increased expression of anti-inflammatory cytokines, such as interleukin (IL)-10, and decreasing expression of pro-inflammatory molecules, e.g., tumor necrosis factor (TNF)-α and IL-6 [79].

The relationship between vitamin D and cardiac function remains highly controversial. Despite the growing body of evidence demonstrating a link between vitamin D deficiency and cardiovascular risk factors, very few studies have examined the association between vitamin D status and cardiac structure and function in healthy athletes. Future research should look to identify the precise mechanisms causing cardiac hypertrophy with increases in vitamin D status in healthy athletes.

### Bone Health and Fracture Risk

Vitamin D status is indicative of calcium absorption and bone mineralization [80], and a considerable expanse of knowledge describes the relationship between vitamin D deficiency and bone health [80–87]. Genetic, environmental, and cultural factors associated with 25[OH]D deficiency [88] increase the risk of osteoporosis [89] and are a major contributor to fracture risk [90]. However, observational studies fail to universally affirm a proportionate susceptibility to bone loss, osteoporotic fractures, or rickets [91, 92], particularly in athletes, a population in which stress fractures are frequently observed [93].

Bone is a metabolically active tissue capable of adapting to mechanical stimuli and repairing structural damage [94]. Bone remodeling is a dynamic physiological process that consists of three main consecutive processes: (1) resorption, when osteoclasts digest old bone; (2) reversal, when mononuclear cells appear on the bone surface; and (3) formation, when osteoblasts lay down new bone until the resorbed bone is completely replaced [95–97]. The regulation of osteoblast function is of greatest relevance to understanding how vitamin D functions in bone. Vitamin D impacts on osteoblast/osteocyte regulation in the process of bone remodeling, and osteoblasts respond to a variety of resorptive signals, including 1,25[OH]_2_D_3_ and parathyroid hormone (PTH). The active form of vitamin D, 1,25[OH]_2_D_3_ affects osteoblast function via different mechanisms. It controls remodeling via induction of receptor activator of nuclear factor (NF)-κB ligand (RANKL) [98], regulates phosphate homeostasis by increasing fibroblast growth factor 23 (FGF23) [99], and may enhance the response of mechanical loads via stimulation of mitogen-activated protein kinase signaling [100]. Evidence now shows that bone cells can produce 1,25[OH]_2_D_3_ from the 25[OH]D_3_ precursor and that this activity is likely to account for the skeletal effects of circulating 25[OH]D_3_ [101].

Athletes undertake mechanical loading from training or competition that is associated with an increase in bone mineral density (BMD) [102, 103]. Any training-induced increase in body mass contributes to the process of bone remodeling and forms mechanically appropriate bone structure [104]. The stimulus of loading the musculoskeletal system through high-intensity dynamic sporting activity is proposed to compensate for 25[OH]D deficiency, with the absence of poor bone health in athletes [102, 105]. However, non-weight-bearing athletes are prone to the same detrimental skeletal effects [104, 106] and are at higher risk for low BMD when vitamin D status is low [107–109].

Recent research shows no association between serum 25[OH]D concentration and measures of bone health in an ethnically diverse athletic population, irrespective of exercise type (weight/non-weight bearing) [110]. This draws into question the use of 25[OH]D concentration as a measure for predicting bone health in the athletic population. Genetic polymorphism in the 25[OH]D/1,25[OH]_2_D pathway may potentially account for some of these differences [22, 111]. This notion is supported by recent research that demonstrated racial differences in manifestations for vitamin D and markers of bone health [112, 113]. Further detail on this phenomenon is described in Sect. 5 of this review.

Optimum concentrations of serum 25[OH]D for the best possible skeletal health are still debated. Many investigators define the threshold for vitamin D sufficiency as the lowest serum 25[OH]D concentration that maximally suppresses PTH secretion and/or optimizes BMD [114–116]. Observational studies have shown inconsistent associations between BMD and serum 25[OH]D status [117, 118], particularly in racial minorities and athletic populations [113, 119, 120]. Further work, including controlled genetic studies, is needed to discriminate between direct actions of 1,25[OH]_2_D_3_ on osteoblasts.

## Vitamin D-Binding Protein (VDBP), Polymorphisms, and the Black Athlete Paradox

There appears to be a paradoxical relationship between ethnicity and vitamin D concentration that has largely been ignored. When examining 25[OH]D deficiency in ethnically diverse populations, studies demonstrate that Black and Hispanic men are at elevated risk of 25[OH]D deficiency but at lower risk of osteoporosis, rapid bone loss, and associated fractures [112, 113] than Caucasians [121]. In Caucasians, BMD significantly decreases as serum 25[OH]D declines, but this is not observed in Black adults [84].

**Fig. 4:**
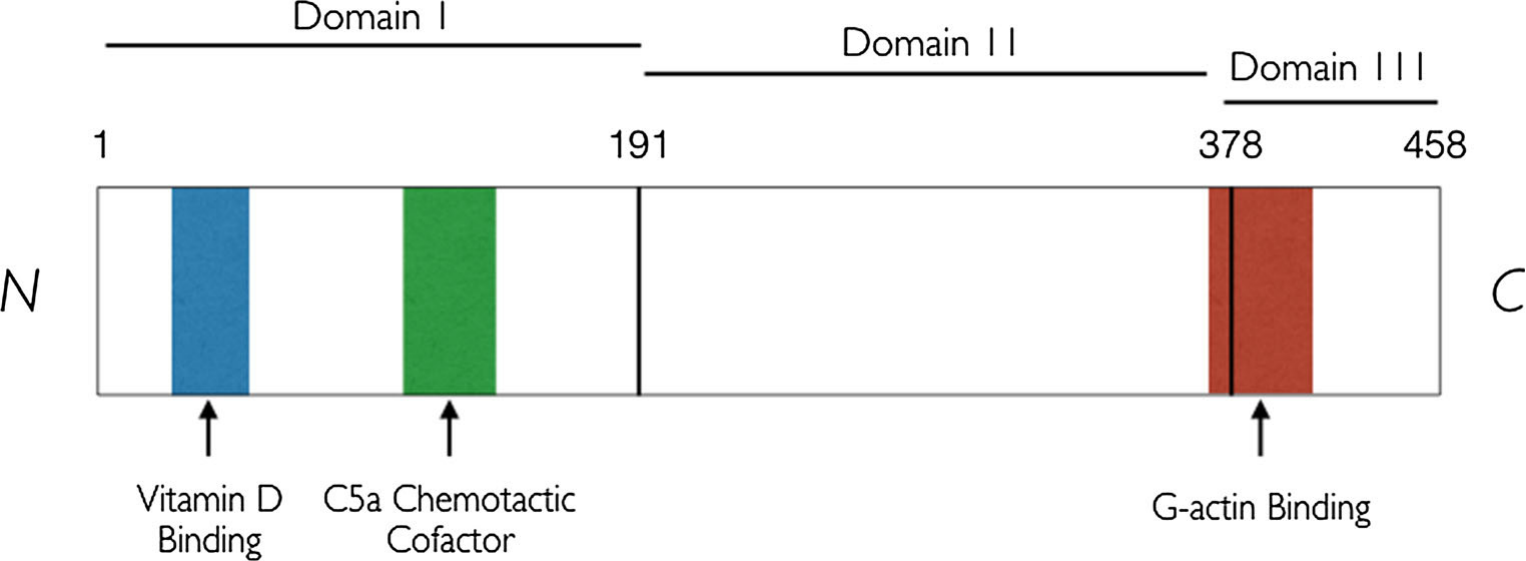
Schematic representation of the vitamin D-binding protein (VDBP) domain structure. The 458 amino acid sequence of human VDBP with the three structural domains and known functional regions is indicated. Domain I: amino acids 1–191; domain II: 192–378; domain III: 379–458; vitamin D binding: 35–49; C5a chemotactic cofactor: 130–149; G-actin binding: 373–403. The domain and functional regions are drawn approximately to scale. The N-terminus refers to the start of a protein or polypeptide terminated by an amino acid with a free amine group (–NH_2_). By convention, peptide sequences are written N-terminus to C-terminus [127]

Vitamin D-binding protein (VDBP) provides insight into why certain ethnic groups may have distinct 25[OH]D and BMD relationships [122]. VDBP is a 51–58 kDa multifunctional and highly polymorphic glycoprotein synthesized primarily by the hepatic parenchymal cells. Originally known as the group-specific component (Gc-globulin), VDBP is a member of a multigene family that includes albumin (Alb) and is a monomeric peptide of 458 residues and three disulphide-bonded, structural domains [123]. Two binding regions have been localized: (1) vitamin D-binding domain, located between residues 35–49 at the N-terminal and (2) actin-binding domain, positioned between residues 350–403 at the C-terminal. These are necessary to mediate VDBP cellular functions [124] (Fig. 4).

VDBP is the primary vitamin D carrier, binding 85–90% of circulating 25[OH]D and 1,25-dihydroxyvitamin D_3_ [1,25[OH]_2_D_3_], the biologically active form of vitamin D, and the remaining unbound 25[OH]D is considered bioavailable (either free or bound to albumin). About 10–15% of total 25[OH]D is bound to albumin, in contrast to free 25[OH]D, which accounts for < 1% of total circulating vitamin D [125]. Since the affinity of albumin to 25[OH]D or 1,25[OH]_2_D_3_ is weaker than that of VDBP, the loosely bound fraction and the free fraction comprise bioavailable 25[OH]D [126].

Genotyping has identified two common single-nucleotide polymorphisms (SNPs) in the coding region of the *VDBP* gene (rs4588 and rs7041) [127]. Combinations of these two SNPs produce three major polymorphic forms of VDBP (Gc1F, Gc1S, and Gc2), which differ substantially in their binding affinity for 25[OH]D, circulating concentration, and variation between ethnic groups [128] and are in turn linked to VDBP function. These variants change the amino acid sequence, alter the protein function, and are common enough to generate population-wide constitutive differences in vitamin D status [50, 127]. Therefore, racial differences in manifestations of vitamin D deficiency may indeed be related to genetic variation in VDBP [128]. However, to date, research on vitamin D status in athletes has overlooked these common allelic variations in VDBP. These findings also question the relative importance of measuring total 25[OH]D vs. the unbound bioavailable fraction of 25[OH]D (discussed in detail in Sect. 7).

## Too Much of a Good Thing

Figure 1 highlights that serum 25[OH]D levels that are too high (> 180 nmol·l^−1^) may be toxic, according to the US IoM. Case reports of vitamin D toxicity are limited, but, nevertheless, there is a risk of toxicity when supplementing with exogenous vitamin D. Despite the search for “optimal” serum 25[OH]D concentrations, very few studies have examined whether high concentrations of 25[OH]D that do not result in toxicity are actually beneficial. Our group recently began to address this question by examining the effects of high-dose vitamin D_3_ supplementation (35,000 IU vs. 70,000 IU weekly) on all major vitamin D metabolites (25[OH]D, 1,25[OH]_2_D_3_, 24[25[OH]D, and PTH) in a cohort of elite athletes [129]. Our findings suggested that both doses effectively raised serum 25[OH]D and 1,25[OH]_2_D_3_; however, the highest dose (70,000 IU/week) also raised the product of vitamin D catabolism, 24,25[OH]D. This metabolite is thought to exert a negative effect on 1,25[OH]_2_D_3_ signaling and may inhibit the conversion of 25[OH]D to 1,25[OH]_2_D_3_ in a negative feedback loop. Interestingly, when athletes were withdrawn from supplementation, the 24,25[OH]D metabolite remained elevated even though 25[OH]D and 1,25[OH]_2_D_3_ fell. One could speculate that persistent elevation of 24,25[OH]D in the face of declining active 1,25[OH]_2_D_3_ could result in the opposite effect than what was intended. Recent evidence supporting the examination of all vitamin D metabolites has emerged. Pleiotropic effects of the vitamin D metabolome were observed in a study of vitamin D status and muscle function and gene expression in the elderly, suggesting that future supplementation studies should not be restricted to usual analysis of the major circulating form of vitamin D, 25[OH]D [15].

## Are We Measuring the Right Thing?

As described earlier, in most clinical and athlete trials, serum 25[OH]D concentration is measured as a marker of vitamin D status because of its long half-life and close relationship to vitamin D_3_ exposure (dermal synthesis or dietary intake). Despite being highly relevant to total and bioavailable vitamin D concentrations, VDBP is not included in most studies examining vitamin D deficiency and measures of health in athletes. Nevertheless, racial differences in VDBP have been explored in the general population to some degree. A recent study demonstrated that community-dwelling Black subjects had lower levels of VDBP and serum 25[OH]D (38.9 ± 0.5 nmol·l^−1^) than White subjects (64.4 ± 0.9 nmol·l^−1^) [122]. However, the authors showed that Black subjects had levels of bioavailable 25[OH]D similar to those of White subjects (2.9 ± 0.1 and 3.1 ± 0.1 ng·ml^−1^, respectively), which may explain why Black subjects presented with consistently lower serum 25[OH]D but higher BMD compared with White subjects [122].

This questions the validity of the commonly used laboratory test for serum 25[OH]D concentrations in assessing vitamin D deficiency in ethnically diverse groups. Consistent with the “free hormone” hypothesis [14], several recent studies have shown that some functions of vitamin D may be more closely related to the free or bioavailable fraction of vitamin D than to total serum 25[OH]D concentrations. For instance, the bioavailable fraction of circulating 25[OH]D was more strongly associated with BMD than the total levels in healthy adults [128]. Similar findings have been observed between bioavailable 25[OH]D and intact PTH, a marker of calcium balance related to bone health [130]. Emerging research also suggests that bioavailable vitamin D is a better predictor of BMD in an ethnically diverse athletic population than serum 25[OH]D concentration [131] and may provide insight into why no universally accepted consensus for vitamin D levels currently exists. For practitioners who wish to measure bioavailable vitamin D, this has been reported to be performed by competitive radioligand-binding assay [128] and antibody-based assays [110], which are commercially available laboratory kits. As with the measurement of total 25[OH]D, specialized laboratory equipment and personnel trained in these techniques are required. No comparison of techniques and establishment of a gold standard have yet been reported.

As our understanding of vitamin D biology develops, it is becoming clearer that determining true vitamin D status is multifactorial. Systematic screening to determine 25[OH]D concentrations in isolation is expensive and demonstrates a poor relationship to bone health. If testing is warranted, practitioners should use the appropriate assays to determine bioavailable (free) vitamin D concentration rather than total serum 25[OH]D and VDBP genotype, if possible.

## Conclusions

**Fig. 5:**
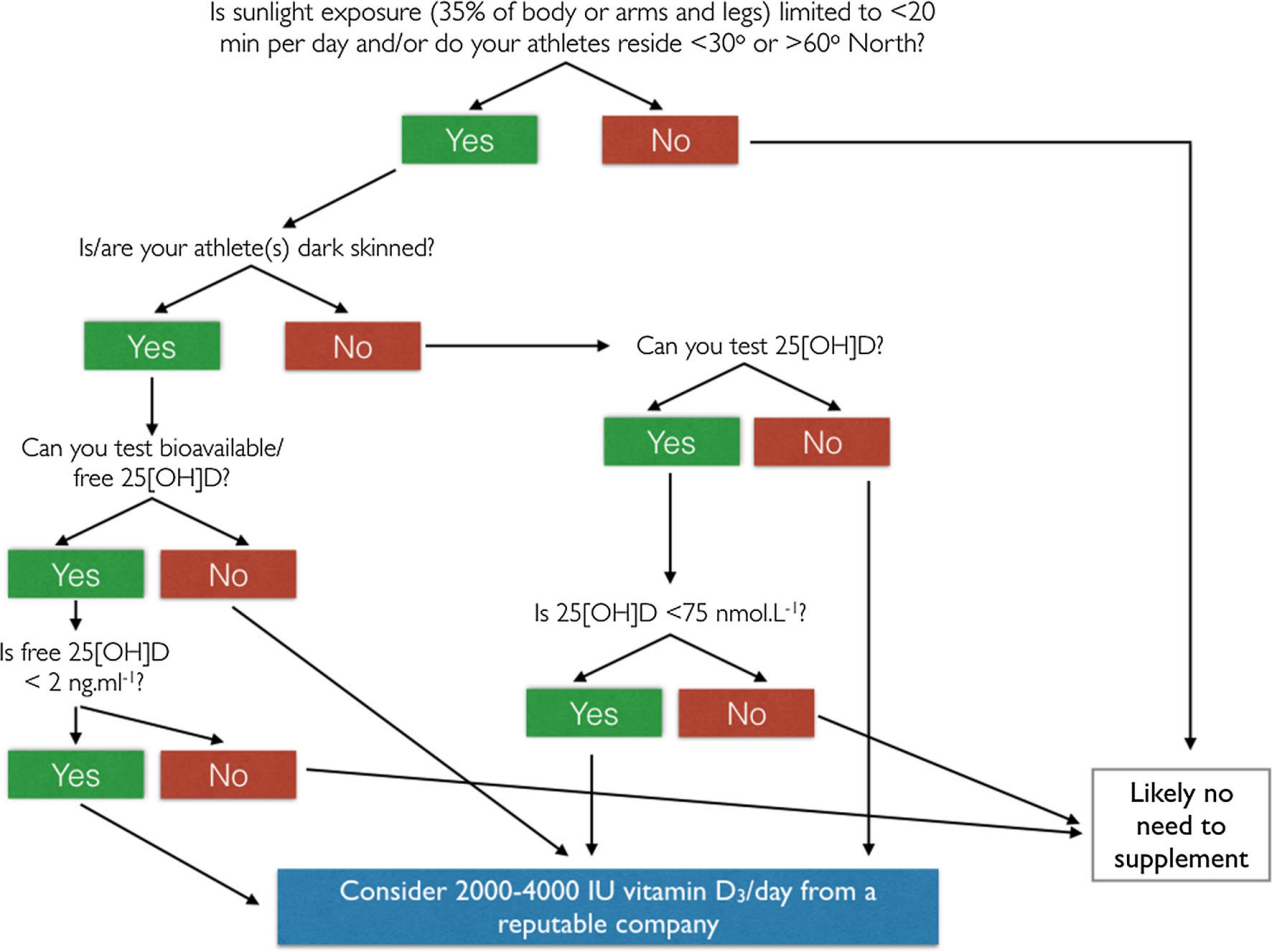
Vitamin D supplementation decision tree for use with athletes

The emerging body of evidence surrounding vitamin D and athletic performance is bolstering support for the need to control vitamin D concentration in athletes. Undoubtedly, adverse risks are associated with vitamin D deficiency that will affect athletic performance directly and indirectly. New insights into the responses of the vitamin D metabolome to supplementation, and emerging evidence suggestive that free 25[OH]D may be a more useful marker of vitamin D status, add new complexities to the area. Nevertheless, the field moves closer to a more complete understanding of the vitamin D endocrine system. The purpose of our review was to collate the most recent advances in this field and provide suggestions, based on current understanding, as to how vitamin D can be managed in practice. To this end, we have structured our thoughts into a decision tree (Fig. 5) that we believe will yield the most effective, safe protocols for those dealing with a broad range of athletes. We hope to inform the field that blanket approaches to supplementation, mega doses of vitamin D, bolus doses of vitamin D, and lack of consideration for an individualized approach should be avoided.

Finally, a note on the future direction of this field. It has recently been proposed that misinterpretation of IoM guidelines has led to the erroneous notion that any population must have a serum concentration above the recommended daily allowance for vitamin D to achieve good bone health (as this is what the dietary reference values for vitamin D have been developed upon) [13]. In addition, because of this misinterpretation, it was suggested that vitamin D deficiency is in fact not epidemic. Although this advice does raise the important point that guidelines should be critically interpreted, it does not consider that vitamin D has pleiotropic effects and may be required in higher quantities in various tissues, as many trials have proposed. Nor does it address the fact that measurement of total serum 25[OH]D is not the most appropriate marker of vitamin D status in ethnically diverse groups. Even employing the correct measurement of free 25[OH]D may be insufficient, as VDBP and VDR phenotype may also affect the responsiveness to vitamin D [14, 132]. This leads us to propose that the field is at a crucial turning point. Before more work and funds are dedicated to identifying what vitamin D targets and how, it is vital to understand the relationship between free 25[OH]D, genotypes in the vitamin D endocrine system, and health. Where such measurements are made, controlling for VDBP and VDR genotype and screening all possible and informative aspects of the vitamin D metabolome will yield the most useful results.

## References

[1] McCollum EV, Simmonds N, Becker JE (1922). Studies on experimental rickets. XXI. An experimental demonstration of the existence of a vitamin which promotes calcium deposition. J Biol Chem.

[2] Li YC, Pirro AE, Amling M (1997). Targeted ablation of the vitamin D receptor: an animal model of vitamin D-dependent rickets type II with alopecia. Proc Natl Acad Sci.

[3] Ebeling PR (2014). Vitamin D and bone health: epidemiologic studies. Bonekey Rep.

[4] Zarei A, Morovat A, Javaid K (2016). Vitamin D receptor expression in human bone tissue and dose-dependent activation in resorbing osteoclasts. Bone Res.

[5] Chun RF, Liu PT, Modlin RL (2014). Impact of vitamin D on immune function: lessons learned from genome-wide analysis. Front Physiol.

[6] Lavie CJ, Dinicolantonio JJ, Milani RV (2013). Vitamin D and cardiovascular health. Circulation.

[7] Girgis CM, Clifton-Bligh RJ, Hamrick MW (2013). The roles of vitamin D in skeletal muscle: form, function, and metabolism. Endocr Rev.

[8] Girgis CM, Mokbel N, Cha KM (2014). The vitamin D receptor (VDR) is expressed in skeletal muscle of male mice and modulates 25-hydroxyvitamin D (25OHD) uptake in myofibers. Endocrinology.

[9] Academies The National, Reference Dietary (2011). Dietary Reference intakes for calcium and vitamin D.

[10] Clemens TL, Adams JS, Nolan JM (1982). Measurement of circulating vitamin D in man. Clin Chim Acta.

[11] Zehnder D, Bland R, Williams MC (2001). Extrarenal expression of 25-hydroxyvitamin d(3)-1 alpha-hydroxylase. J Clin Endocrinol Metab.

[12] Snellman G, Melhus H, Gedeborg R (2010). Determining vitamin D status: a comparison between commercially available assays. PLoS One.

[13] Manson JE, Brannon PM, Rosen CJ (2016). Vitamin D deficiency—is there really a pandemic?. N Engl J Med.

[14] Chun RF, Peercy BE, Orwoll ES (2014). Vitamin D and DBP: the free hormone hypothesis revisited. J Steroid Biochem Mol Biol.

[15] Hassan-Smith ZK, Jenkinson C, Smith DJ (2017). 25-Hydroxyvitamin D_3_ and 1,25-dihydroxyvitamin D_3_ exert distinct effects on human skeletal muscle function and gene expression. PLoS One.

[16] Heaney RP, Holick MF (2011). Why the IOM recommendations for vitamin D are deficient. J Bone Miner Res.

[17] Zittermann A (2003). Vitamin D in preventive medicine: are we ignoring the evidence?. Br J Nutr.

[18] Backx E, van der Avoort C, Tieland M (2017). Seasonal variation in vitamin D status in elite athletes: a longitudinal study. Int J Sport Nutr Exerc Metab.

[19] Bescos-Garcia R, Rodriguez-Guisado FA (2011). Low levels of vitamin D in professional basketball players after wintertime: relationship with dietary intake of vitamin D and calcium. Nutr Hosp.

[20] Fishman MP, Lombardo SJ, Kharrazi FD (2016). Vitamin D deficiency among professional basketball players. Orthop J Sports Med.

[21] Flueck JL, Hartmann K, Strupler M (2016). Vitamin D deficiency in Swiss elite wheelchair athletes. Spinal Cord.

[22] Hamilton B, Grantham J, Racinais S (2010). Vitamin D deficiency is endemic in Middle Eastern sportsmen. Public Health Nutr.

[23] Krzywanski J, Mikulski T, Krysztofiak H (2016). Seasonal vitamin D status in Polish elite athletes in relation to sun exposure and oral supplementation. PLoS One.

[24] Magee PJ, Pourshahidi LK, Wallace JM (2013). Vitamin D status and supplementation in elite irish athletes. Int J Sport Nutr Exerc Metab.

[25] Morton JP, Iqbal Z, Drust B (2012). Seasonal variation in vitamin D status in professional soccer players of the English Premier League. Appl Physiol Nutr Metab.

[26] Pritchett K, Pritchett R, Ogan D (2016). 25(OH)D status of elite athletes with spinal cord injury relative to lifestyle factors. Nutrients.

[27] Sghaier-Ayadi A, Feki M, Ayed IB (2015). Vitamin D status and determinants of deficiency in non-supplemented athletes during the winter months in Tunisia. Biol Sport.

[28] Valtuena J, Dominguez D, Til L (2014). High prevalence of vitamin D insufficiency among elite Spanish athletes the importance of outdoor training adaptation. Nutr Hosp.

[29] Close GL, Russell J, Cobley JN (2013). Assessment of vitamin D concentration in non-supplemented professional athletes and healthy adults during the winter months in the UK: implications for skeletal muscle function. J Sports Sci.

[30] Chen T, Chimeh F, Lu Z (2007). Factors that influence the cutaneous synthesis and dietary sources of vitamin D. Arch Bochem Biophys.

[31] Owens DJ, Sharples AP, Polydorou I (2015). A systems based investigation into vitamin D and skeletal muscle repair, regeneration and hypertrophy. Am J Physiol.

[32] Barker T, Henriksen VT, Martins TB (2013). Higher serum 25-hydroxyvitamin D concentrations associate with a faster recovery of skeletal muscle strength after muscular injury. Nutrients.

[33] Barker T, Schneider ED, Dixon BM (2013). Supplemental vitamin D enhances the recovery in peak isometric force shortly after intense exercise. Nutr Metab.

[34] Agergaard J, Trostrup J, Uth J (2015). Does vitamin-D intake during resistance training improve the skeletal muscle hypertrophic and strength response in young and elderly men? A randomized controlled trial. Nutr Metab.

[35] Chaillou T, Lee JD, England JH (2013). Time course of gene expression during mouse skeletal muscle hypertrophy. J Appl Physiol.

[36] Olsson K, Saini A, Stromberg A (2016). Evidence for vitamin D receptor expression and direct effects of 1alpha,25(OH)2D_3_ in human skeletal muscle precursor cells. Endocrinology.

[37] Conboy IM, Rando TA (2002). The regulation of Notch signaling controls satellite cell activation and cell fate determination in postnatal myogenesis. Dev Cell.

[38] Domingues-Faria C, Chanet A, Salles J (2014). Vitamin D deficiency down-regulates Notch pathway contributing to skeletal muscle atrophy in old Wistar rats. Nutr Metab.

[39] Glass DJ (2010). PI3 kinase regulation of skeletal muscle hypertrophy and atrophy. Curr Top Microbiol Immunol.

[40] Stewart R, Flechner L, Montminy M (2011). CREB is activated by muscle injury and promotes muscle regeneration. PLoS One.

[41] Wyon MA, Koutedakis Y, Wolman R (2014). The influence of winter vitamin D supplementation on muscle function and injury occurrence in elite ballet dancers: a controlled study. J Sci Med Sport.

[42] Hamilton B, Whiteley R, Farooq A (2014). Vitamin D concentration in 342 professional football players and association with lower limb isokinetic function. J Sci Med Sport.

[43] Owens DJ, Webber D, Impey SG (2014). Vitamin D supplementation does not improve human skeletal muscle contractile properties in insufficient young males. Eur J Appl Physiol.

[44] Stockton KA, Mengersen K, Paratz JD (2011). Effect of vitamin D supplementation on muscle strength: a systematic review and meta-analysis. Osteoporos Int.

[45] Janssen HC, Samson MM, Verhaar HJ (2002). Vitamin D deficiency, muscle function, and falls in elderly people. Am J Clin Nutr.

[46] He CS, Aw Yong XH, Walsh NP (2016). Is there an optimal vitamin D status for immunity in athletes and military personnel?. Exerc Immunol Rev.

[47] Hossein-Nezhad A, Holick MF (2013). Vitamin D for health: a global perspective. Mayo Clin Proc.

[48] Bikle DD (2009). Vitamin D and immune function: understanding common pathways. Curr Osteoporos Rep.

[49] Liu PT, Stenger S, Li H (2006). Toll-like receptor triggering of a vitamin D-mediated human antimicrobial response. Science.

[50] Wang TJ, Pencina MJ, Booth SL (2008). Vitamin D deficiency and risk of cardiovascular disease. Circulation.

[51] Gombart AF, Borregaard N, Koeffler HP (2005). Human cathelicidin antimicrobial peptide (CAMP) gene is a direct target of the vitamin D receptor and is strongly up-regulated in myeloid cells by 1,25-dihydroxyvitamin D_3_. FASEB J.

[52] Cannell JJ, Hollis BW (2008). Use of vitamin D in clinical practice. Altern Med Rev.

[53] Cannell JJ, Vieth R, Umhau JC (2006). Epidemic influenza and vitamin D. Epidemiol Infect.

[54] Urashima M, Segawa T, Okazaki M (2010). Randomized trial of vitamin D supplementation to prevent seasonal influenza A in schoolchildren. Am J Clin Nutr.

[55] Hewison M (2012). An update on vitamin D and human immunity. Clin Endocrinol.

[56] Al-Jaderi Z, Maghazachi AA (2013). Effects of vitamin D_3_, calcipotriol and FTY720 on the expression of surface molecules and cytolytic activities of human natural killer cells and dendritic cells. Toxins.

[57] Sly LM, Lopez M, Nauseef WM (2001). 1alpha,25-Dihydroxyvitamin D_3_-induced monocyte antimycobacterial activity is regulated by phosphatidylinositol 3-kinase and mediated by the NADPH-dependent phagocyte oxidase. J Biol Chem.

[58] Cox AJ, Gleeson M, Pyne DB (2008). Clinical and laboratory evaluation of upper respiratory symptoms in elite athletes. Clin J Sport Med.

[59] Laaksi I, Ruohola JP, Tuohimaa P (2007). An association of serum vitamin D concentrations <40 nmol/L with acute respiratory tract infection in young Finnish men. Am J Clin Nutr.

[60] Berry DJ, Hesketh K, Power C (2011). Vitamin D status has a linear association with seasonal infections and lung function in British adults. Br J Nutr.

[61] Ginde AA, Mansbach JM, Camargo CA (2009). Association between serum 25-hydroxyvitamin D level and upper respiratory tract infection in the Third National Health and Nutrition Examination Survey. Arch Intern Med.

[62] Sabetta JR, DePetrillo P, Cipriani RJ (2010). Serum 25-hydroxyvitamin D and the incidence of acute viral respiratory tract infections in healthy adults. PLoS One.

[63] Halliday TM, Peterson NJ, Thomas JJ (2011). Vitamin D status relative to diet, lifestyle, injury, and illness in college athletes. Med Sci Sports Exerc.

[64] He CS, Handzlik M, Fraser WD (2013). Influence of vitamin D status on respiratory infection incidence and immune function during 4 months of winter training in endurance sport athletes. Exerc Immunol Rev.

[65] He CS, Fraser WD, Tang J (2016). The effect of 14 weeks of vitamin D_3_ supplementation on antimicrobial peptides and proteins in athletes. J Sports Sci.

[66] Chen S, Glenn DJ, Ni W (2008). Expression of the vitamin D receptor is increased in the hypertrophic heart. Hypertension.

[67] Weishaar RE, Simpson RU (1987). Vitamin D_3_ and cardiovascular function in rats. J Clin Investig.

[68] Achinger SG, Ayus JC (2005). The role of vitamin D in left ventricular hypertrophy and cardiac function. Kidney Int.

[69] Giovannucci E, Liu Y, Hollis BW (2008). 25-hydroxyvitamin D and risk of myocardial infarction in men: a prospective study. Arch Intern Med.

[70] Scragg R, Jackson R, Holdaway IM (1990). Myocardial infarction is inversely associated with plasma 25-hydroxyvitamin D_3_ levels; a community based study. Int J Epidemiol.

[71] Kamycheva E, Johnsen SH, Wilsgaard T (2013). Evaluation of serum 25-hydroxyvitamin D as a predictor of carotid intima-media thickness and carotid total plaque area in nonsmokers: the Tromso study. Int J Endocrinol.

[72] Pilz S, Tomaschitz A, Marz W (2011). Vitamin D, cardiovascular disease and mortality. Clin Endocrinol.

[73] Akin F, Ayca B, Kose N (2014). Serum vitamin D and C-reactive protein levels are independently associated with diastolic dysfunction. J Investig Med.

[74] Weiner RB, Baggish AL (2012). Exercise-induced cardiac remodeling. Prog Cardiovasc Dis.

[75] Constantini NW, Arieli R, Chodick G (2010). High prevalence of vitamin D insufficiency in athletes and dancers. Clin J Sport Med.

[76] Allison RJ, Close GL, Farooq A (2015). Severely vitamin D-deficient athletes present smaller hearts than sufficient athletes. Eur J Prev Cardiol.

[77] Andrukhova O, Slavic S, Zeitz U (2014). Vitamin D is a regulator of endothelial nitric oxide synthase and arterial stiffness in mice. Mol Endocrinol.

[78] Raymond MA, Desormeaux A, Labelle A (2005). Endothelial stress induces the release of vitamin D-binding protein, a novel growth factor. Biochem Biophys Res Commun.

[79] Zittermann A, Schleithoff SS, Koerfer R (2005). Putting cardiovascular disease and vitamin D insufficiency into perspective. Br J Nutr.

[80] Berry JL, Davies M, Mee AP (2002). Vitamin D metabolism, rickets, and osteomalacia. Semin Musculoskelet Radiol.

[81] Breen ME, Laing EM, Hall DB (2010). 25-Hydroxyvitamin D, insulin-like growth factor-1, and bone mineral accrual during growth. J Clin Endocrinol Metab.

[82] Cashman KD, Hill TR, Cotter AA (2008). Low vitamin D status adversely affects bone health parameters in adolescents. Am J Clin Nutr.

[83] Collins D, Jasani C, Fogelman I (1998). Vitamin D and bone mineral density. Osteoporos Int.

[84] Gutierrez OM, Farwell WR, Kermah D (2011). Racial differences in the relationship between vitamin D, bone mineral density, and parathyroid hormone in the National Health and Nutrition Examination Survey. Osteoporos Int.

[85] Holick MF (2006). Resurrection of vitamin D deficiency and rickets. J Clin Investig.

[86] Sadat-Ali M, Al Elq AH, Al-Turki HA (2011). Influence of vitamin D levels on bone mineral density and osteoporosis. Ann Saudi Med.

[87] Wöfl C, Englert S, Moghaddam AA (2013). Time course of 25(OH)D_3_ vitamin D_3_ as well as PTH (parathyroid hormone) during fracture healing of patients with normal and low bone mineral density (BMD). BMC Musculoskelet Disord.

[88] Kanis JA (2002). Diagnosis of osteoporosis and assessment of fracture risk. Lancet.

[89] Allali F, El Aichaoui S, Saoud B (2006). The impact of clothing style on bone mineral density among post menopausal women in Morocco: a case–control study. BMC Public Health.

[90] Dhesi JK (2004). Vitamin D supplementation improves neuromuscular function in older people who fall. Age Ageing.

[91] Hamson C, Goh L, Sheldon P (2003). Comparative study of bone mineral density, calcium, and vitamin D status in the Gujarati and white populations of Leicester. Postgrad Med J.

[92] Lowe NM, Mitra SR, Foster PC (2010). Vitamin D status and markers of bone turnover in Caucasian and South Asian postmenopausal women living in the UK. Br J Nutr.

[93] Johnson AW, Weiss CB, Wheeler DL (1994). Stress fractures of the femoral shaft in athletes–more common than expected. A new clinical test. Am J Sports Med..

[94] Frost HM (1969). Tetracycline-based histological analysis of bone remodeling. Calcif Tissue Res.

[95] Ashman RB, Van Buskirk WC, Cowin SC (1985). The mechanical properties of immature osteopetrotic bone. Calcif Tissue Int.

[96] Cowin SC (1993). Bone stress adaptation models. J Biomech Eng.

[97] Hadjidakis DJ, Androulakis II (2006). Bone remodeling. Ann N Y Acad Sci.

[98] Kim S, Yamazaki M, Zella LA (2006). Activation of receptor activator of NF-kappaB ligand gene expression by 1,25-dihydroxyvitamin D_3_ is mediated through multiple long-range enhancers. Mol Cell Biol.

[99] Shimada T, Hasegawa H, Yamazaki Y (2004). FGF-23 is a potent regulator of vitamin D metabolism and phosphate homeostasis. J Bone Miner Res.

[100] Robling AG, Castillo AB, Turner CH (2006). Biomechanical and molecular regulation of bone remodeling. Annu Rev Biomed Eng.

[101] Anderson PH, Atkins GJ (2008). The skeleton as an intracrine organ for vitamin D metabolism. Mol Aspects Med.

[102] Nikander R, Sievanen H, Uusi-Rasi K (2006). Loading modalities and bone structures at nonweight-bearing upper extremity and weight-bearing lower extremity: a pQCT study of adult female athletes. Bone.

[103] Rantalainen T, Nikander R, Daly RM (2011). Exercise loading and cortical bone distribution at the tibial shaft. Bone.

[104] Nikander R, Sievanen H, Heinonen A (2005). Femoral neck structure in adult female athletes subjected to different loading modalities. J Bone Miner Res.

[105] Weidauer L, Minett M, Negus C (2014). Odd-impact loading results in increased cortical area and moments of inertia in collegiate athletes. Eur J Appl Physiol.

[106] Fredericson M, Chew K, Ngo J (2007). Regional bone mineral density in male athletes: a comparison of soccer players, runners and controls. Br J Sports Med.

[107] Guillaume G, Chappard D, Audran M (2012). Evaluation of the bone status in high-level cyclists. J Clin Densitom.

[108] Rector RS, Rogers R, Ruebel M (2008). Participation in road cycling vs running is associated with lower bone mineral density in men. Metabolism.

[109] Smathers AM, Bemben MG, Bemben DA (2009). Bone density comparisons in male competitive road cyclists and untrained controls. Med Sci Sports Exerc.

[110] Allison RJ, Farooq A, Hamilton B (2016). No association between vitamin D deficiency and markers of bone health in athletes. Med Sci Sports Exerc.

[111] Rabon-Stith KM, Hagberg JM, Phares DA (2005). Vitamin D receptor FokI genotype influences bone mineral density response to strength training, but not aerobic training. Exp Physiol.

[112] Cauley JA, Lui LY, Ensrud KE (2005). Bone mineral density and the risk of incident nonspinal fractures in black and white women. JAMA.

[113] Hannan MT, Litman HJ, Araujo AB (2008). Serum 25-hydroxyvitamin D and bone mineral density in a racially and ethnically diverse group of men. J Clin Endocrinol Metab.

[114] Bischoff-Ferrari HA, Giovannucci E, Willett WC (2006). Estimation of optimal serum concentrations of 25-hydroxyvitamin D for multiple health outcomes. Am J Clin Nutr.

[115] Chapuy MC, Preziosi P, Maamer M (1997). Prevalence of vitamin D insufficiency in an adult normal population. Osteoporos Int.

[116] Malabanan A, Veronikis IE, Holick MF (1998). Redefining vitamin D insufficiency. Lancet.

[117] Bischoff-Ferrari HA, Kiel DP, Dawson-Hughes B (2009). Dietary calcium and serum 25-hydroxyvitamin D status in relation to BMD among U.S. adults. J Bone Miner Res.

[118] Marwaha RK, Tandon N, Garg MK (2011). Bone health in healthy Indian population aged 50 years and above. Osteoporos Int.

[119] Gerdhem P, Ringsberg KAM, Obrant KJ (2005). Association between 25-hydroxy vitamin D levels, physical activity, muscle strength and fractures in the prospective population-based OPRA study of elderly women. Osteoporos Int.

[120] Kremer R, Campbell PP, Reinhardt T (2009). Vitamin D status and its relationship to body fat, final height, and peak bone mass in young women. J Clin Endocrinol Metab.

[121] Engelman CD, Fingerlin TE, Langefeld CD (2008). Genetic and environmental determinants of 25-hydroxyvitamin D and 1,25-dihydroxyvitamin D levels in Hispanic and African Americans. J Clin Endocrinol Metab.

[122] Powe CE, Evans MK, Wenger J (2013). Vitamin D-binding protein and vitamin D status of black Americans and white Americans. N Engl J Med.

[123] Haddad JG, Hu YZ, Kowalski MA (1992). Identification of the sterol- and actin-binding domains of plasma vitamin D binding protein (Gc-globulin). Biochemistry.

[124] Zhang J, Habiel DM, Ramadass M (2010). Identification of two distinct cell binding sequences in the vitamin D binding protein. Biochim Biophys Acta.

[125] Bikle DD, Gee E, Halloran B (1986). Assessment of the free fraction of 25-hydroxyvitamin D in serum and its regulation by albumin and the vitamin D-binding protein. J Clin Endocrinol Metab.

[126] Brown AJ, Coyne DW (2012). Bioavailable vitamin D in chronic kidney disease. Kidney Int.

[127] Malik S, Fu L, Juras DJ (2013). Common variants of the vitamin D binding protein gene and adverse health outcomes. Crit Rev Clin Lab Sci.

[128] Powe CE, Ricciardi C, Berg AH (2011). Vitamin D-binding protein modifies the vitamin D-bone mineral density relationship. J Bone Miner Res.

[129] Owens DJ, Tang JC, Bradley WJ (2017). Efficacy of high-dose vitamin D supplements for elite athletes. Med Sci Sports Exerc.

[130] Shieh A, Chun RF, Ma C (2016). Effects of high-dose vitamin D_2_ versus D_3_ on total and free 25-hydroxyvitamin D and markers of calcium balance. J Clin Endocrinol Metab.

[131] Allison RJ, Farooq A, Cherif A, et al. Why don't serum vitamin D concentrations associate with BMD by DXA? A case of being 'bound' to the wrong assay? Implications for vitamin D screening. Br J Sports Med. 2017. 10.1136/bjsports-2016-097130 10.1136/bjsports-2016-097130 28798036

[132] Sollid ST, Hutchinson MY, Berg V (2016). Effects of vitamin D binding protein phenotypes and vitamin D supplementation on serum total 25(OH)D and directly measured free 25(OH)D. Eur J Endocrinol.

[133] Papadakis M, Wilson MG, Ghani S (2012). Impact of ethnicity upon cardiovascular adaptation in competitive athletes: relevance to preparticipation screening. Br J Sports Med.

